# Effect of Foot Orthoses and Footwear in People with Rheumatoid Arthritis: An Updated Systematic Review

**DOI:** 10.3390/healthcare12202017

**Published:** 2024-10-11

**Authors:** José Manuel Cabrera-Sánchez, María Reina-Bueno, Inmaculada C. Palomo-Toucedo, María del Carmen Vázquez-Bautista, María-Ángeles Núñez-Baila, José Rafael González-López

**Affiliations:** 1 Department of Podiatry, Faculty of Nursing, Physiotherapy and Podiatry, Universidad de Sevilla, 41009 Seville, Spain; joscabsan3@alum.us.es (J.M.C.-S.); ipalomo@us.es (I.C.P.-T.); carmenvaz@us.es (M.d.C.V.-B.); 2 Department of Nursing, Faculty of Nursing, Physiotherapy and Podiatry, Universidad de Sevilla, 41009 Seville, Spain; mnbaila@us.es (M.-Á.N.-B.); joserafael@us.es (J.R.G.-L.); 3 Instituto de Biomedicina de Sevilla, IBiS/Hospital Universitario Virgen del Rocío/CSIC/Universidad de Sevilla, 41013 Seville, Spain

**Keywords:** foot deformity, foot function, foot pain, inflammatory arthritis, lower-limb orthoses, shoes

## Abstract

Background/Objectives: Rheumatoid arthritis is a chronic, systemic, inflammatory disease of an autoimmune nature that causes pain and disability in affected patients. Foot pain has become a challenge due to its negative impact on physical function. The objective of this updated systematic review is to describe the effect of foot orthoses and/or footwear in patients with rheumatoid arthritis and foot problems. Methods: Scopus, PubMed, CINALH, WOS, and Dialnet were searched for all articles published from January 2013 to September 2024. Inclusion criteria included randomised clinical trials and crossover trials (level of evidence I), published within the last 10 years, involving adults with a diagnosis of rheumatoid arthritis, with no restrictions on gender, race, or ethnicity. All studies that addressed the use of foot orthoses and/or shoe therapy in any type of comparison between these interventions were considered relevant. Review Manager was used to carry out the bias analysis of the selected studies. The reporting was based on the new PRISMA guidelines. Results: A total of 9 relevant articles were selected from an initial sample of 438. These articles analyse and compare the effectiveness of various types of foot orthoses in reducing pain, functional limitation, and disability, as well as improving balance and kinetic and kinematic parameters affected by rheumatoid arthritis. Conclusions: Foot orthoses reduce pain and disability in rheumatoid arthritis, improving balance and kinematic parameters. However, no significant improvements in the patients’ functionality and walking ability have been demonstrated. Customised ones with good arch control, heel reinforcement, and metatarsal pad are more effective. No results on the impact of footwear on patients with rheumatoid arthritis have been found in the last 10 years. This systematic review was registered in PROSPERO (CRD42023405645).

## 1. Introduction

Rheumatoid arthritis (RA) is a chronic autoimmune disease of unknown origin, characterised by inflammation of the joints [1]. The disease progresses in a symmetrical manner, initially affecting small joints and gradually spreading to larger ones [2,3,4]. In addition to its impact on the musculoskeletal system, RA can also lead to extra-articular manifestations [5]. Foot involvement represents a significant clinical challenge in patients with RA, with symptom severity varying based on the duration and progression of the disease [6,7,8,9,10]. The feet are typically more severely affected during the acute phase, with common deformities, such as hallux abductus valgus (HAV), metatarsus primus varus, hallux rigidus, hammertoes, claw toes, flat feet, and a triangular forefoot, frequently observed [6,7,11,12,13,14].

The patient’s gait is significantly altered due to pain, resulting in reduced stride length, gait velocity, cadence, and ankle strength [15,16,17,18]. From a kinematic perspective, there is a notable reduction in maximal ankle plantar flexor power, diminished ankle joint range of motion, and decreased angular velocity [18]. This gait disturbance, coupled with foot pain caused by typical RA deformities, leads to a decline in functional capacity and negatively impacts the patient’s quality of life [7,12,13].

Despite the benefits of pharmacological therapy, it must be complemented by other forms of treatment to achieve more satisfactory outcomes. This leads to the concept of stepped care, which consists of [19] (i) advice on over-the-counter shoes, (ii) ready-made foot orthoses (FOs), (iii) custom-made FOs, and (iv) therapeutic shoes. FOs are considered the first line of conservative treatment for individuals with RA experiencing foot pain. FOs are therapeutic medical devices inserted into footwear with the aim of supporting, preventing, aligning, and treating foot deformities and misalignments. The type of FOs varies depending on several factors, such as the materials used, the design and construction, level of customisation, and the stage and severity of the disease [20,21].

As a precedent of this updated systematic review, the comprehensive systematic review and meta-analysis conducted by Conceição et al. [22] evaluated the effects of FOs on pain and disability in patients with RA. Their findings concluded that while FOs are effective in reducing pain, they do not significantly influence disability outcomes. As such, there remains no consensus among rehabilitation professionals regarding the overall efficacy of FOs in improving both pain and disability in RA patients. Although FOs are suggested to alleviate pain, their impact on reducing disability appears limited. This updated systematic review, covering studies published from January 2013 to September 2024, builds upon the foundation provided by the previous review. Consequently, our study focuses on more recent research to build upon and complement these earlier findings.

The characteristics of the insole primarily depend on the therapeutic objective to be achieved, with satisfactory outcomes reported for FOs featuring semi-rigid covers and soft material in the forefoot, along with the incorporation of a medial wedge at the heel in more severe cases [20,23]. When this treatment proves insufficient, it is appropriate to consider footwear therapy, which may be used either alone or in conjunction with plantar support [24,25]. This type of footwear is designed with specific features to accommodate deformities. Ideally, it should be extra-deep, shock-absorbing, and equipped with wide toe caps to adequately accommodate both the deformities and the FOs, if required [23,24,25].

In this article, we conduct a systematic review of studies focusing on the efficacy of treatment of foot pain in patients with RA using FOs and footwear therapy.

## 2. Materials and Methods

### 2.1. Protocol and Registration

The systematic review conducted here adhered to the Preferred Reporting Items for Systematic Reviews and Meta-Analyses (PRISMA) guidelines [26,27] (Supplementary Material). The study has been duly registered with PROSPERO, the International Prospective Register of Systematic Reviews (Registration ID: CRD42023405645).

### 2.2. Search Strategy

To conduct this review, an extensive search was carried out between March and May 2023 in several databases, including PubMed, Scopus, CINALH, Web of Science, Dialnet, and the FAMA catalogue of the University of Seville. In order to ensure the inclusion of the most recent evidence, an updated search was conducted on 2 October 2024, covering publications from January 2023 to September 2024. Despite this additional search, no new studies meeting the inclusion criteria were found. To optimise the search, health-related descriptors relevant to the subject were used, along with free terms combined with Boolean operators, and in some cases, truncation terms. An advanced search tool was employed in all cases to design the search strategies. A specific strategy was developed for each database, which is detailed in Appendix A.

The aim of this review was to assess the existing evidence on the outcomes of this type of treatment. A PICO (Patient, Intervention, Comparison, Outcome) framework was employed to define the research question, focusing on the clinical intervention and the target population: “How does the use of foot orthoses (FOs) and/or footwear therapy affect patients with rheumatoid arthritis (RA) who present with foot disorders?”

### 2.3. Selection Criteria and Study Selection

Initially, the selection was limited to randomised clinical trials (RCTs) published in English or Spanish with a filter for the past 10 years. Due to the scarcity of studies that met these criteria, the selection criteria were broadened to include crossover trials published in peer-reviewed journals, provided they offered sufficient statistical detail, such as means, standard deviations, and participant numbers for quantitative synthesis. This modification allowed the inclusion of studies with robust designs that were previously excluded, thus enhancing the scope and comprehensiveness of the review. Furthermore, following the updated search conducted in October 2024, the selection criteria were adjusted to focus on RCTs and crossover trials published between January 2023 and September 2024 in English or Spanish.

Thus, inclusion criteria for this review encapsulated the following: (1) RCTs and crossover trials published in peer-reviewed journals in English or Spanish from January 2013 to September 2024, providing sufficient statistical detail, such as means, standard deviations, and participant numbers for quantitative synthesis; (2) interventions that included FOs and/or footwear therapy; (3) study populations that included adult participants without restrictions on gender, race, or ethnicity; (4) participants diagnosed with RA and feet problems. Exclusion criteria comprised the following: (1) articles that were reviews, medical protocols, conference proceedings, case reports, letters, editorials, pilot RCTs, and pilot studies; (2) the results of those studies were not related to the objectives of this review; (3) studies without any comparison group or those not reporting any comparative results between groups; (4) observational studies.

For the study selection process, two independent reviewers, J.M.C.-S. and M.R.-B., were appointed. Each reviewer conducted the selection process independently. In the initial stage, they screened the titles and abstracts of the shortlisted articles. Following this, both reviewers individually assessed whether the studies met the inclusion and exclusion criteria. Additionally, they separately evaluated the key characteristics of the studies to ensure adherence to the defined eligibility criteria.

In instances where disagreements arose regarding the inclusion of specific articles, a third reviewer (M.d.C.V.-B.) was consulted to make the final determination on the suitability of the article in question.

### 2.4. Data Extraction

To facilitate data extraction from the articles included in this review, an Excel spreadsheet was created to systematically organise the information from each study. The inclusion or exclusion of each study was documented, along with the justification for the decision, using a coding system based on the predefined criteria. Each reviewer maintained their own worksheet. Following the final assessment, the results were consolidated, and any discrepancies regarding article inclusion or exclusion were discussed. Initially, these discussions took place between the two primary reviewers, but in some cases, the involvement of a third reviewer was required to reach a final decision.

Ultimately, a single reviewer (J.M.C.-S.) performed the complete data extraction for the articles selected after the second assessment. For this task, a Word document was used to record the identification and key characteristics of the studies (study type, number of participants, mean age, intervention, outcomes, etc.), ensuring strict adherence to the eligibility criteria established for the review

### 2.5. Main Outcome Variables

The primary outcome was the effect of the intervention on pain, disability, and functional limitation. Secondary outcomes included effects of the intervention on individual components of compassion fatigue, including burnout and STS, and other outcomes reported in the individual studies.

The primary outcomes were pain [28,29,30,31,32,33,34], as well as disability and functional limitation [28,29,30,32,33,34]. Secondary outcomes included biomechanical aspects [20,28,29,30,31,32], the self-reported effect of wearing foot orthoses [35], and health-related quality of life [33,34].

### 2.6. Risk of Bias Assessment

To carry out the bias analysis of the selected studies, the tool provided by the Cochrane Collaboration, known as Review Manager (RevMan) web version, was used. This tool can be accessed through the following link: https://revman.cochrane.org/#/myReviews (accessed on 31 May 2023) [36]. Each reviewer independently conducted a thorough assessment of the risk of bias and reassessed whether the articles met the previously established inclusion criteria. Only those studies that satisfied these criteria were ultimately selected for review. In cases where there were disagreements between reviewers, these were resolved through discussion, and if consensus could not be reached, a third reviewer was consulted to ensure an unbiased decision.

The following types of biases were evaluated [37]:

Selection bias—this bias evaluates whether the allocation of participants to the different intervention groups was carried out randomly and whether allocation concealment was implemented to ensure that investigators were blinded to group assignments.

Performance bias—this bias assesses the level of blinding among both participants and personnel involved in conducting the study, ensuring that neither party influenced the outcomes due to knowledge of group assignments.

Detection bias—this involves assessing the blinding of outcome assessors, describing any measures taken to ensure blinding and prevent bias during the evaluation of study results.

Attrition bias—this bias looks at the reporting of dropouts, exclusions, and the number of participants in each intervention group, including the reasons for exclusions, as well as any potential re-inclusion of data in the analyses conducted by the reviewers.

Reporting bias (publication bias)—this bias assesses the possibility of selective reporting of outcomes and the findings that were derived from this evaluation, ensuring that all relevant results were transparently reported.

Other biases—any additional concerns regarding biases that were not covered by the above categories were also considered. This includes biases related to deviations from the study protocol, if applicable, and any other concerns highlighted during the review process.

By evaluating these types of bias, we ensured a comprehensive assessment of the methodological quality of the studies included in this review. This process improves the reliability and validity of the conclusions drawn from the selected studies.

## 3. Results

### 3.1. Study Selection

**Figure 1:**
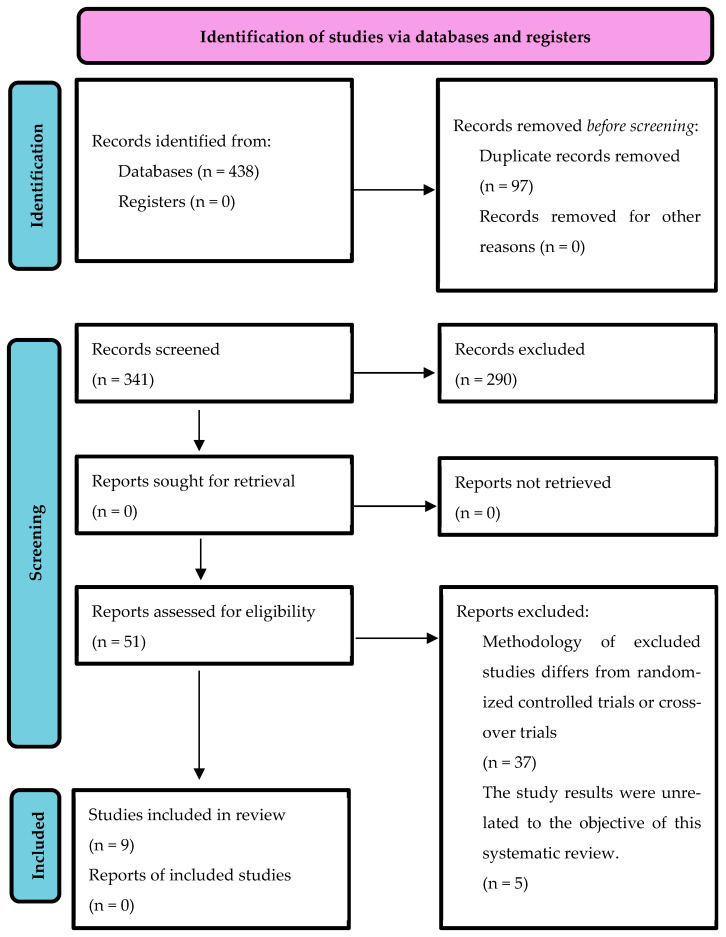
Flow diagram of the study selection.

A total of 438 articles were identified through the search, with 97 duplicates removed using Zotero 7.0. After screening the titles and abstracts, a further 290 articles were excluded. Full-text screening of the remaining 51 studies was then conducted, resulting in the final inclusion of relevant articles. In cases where discrepancies arose between the two researchers, they discussed the articles to reach a consensus. Following the full-text review, 42 studies were rejected: 37 were not RCTs, and 5 contained irrelevant information. Figure 1 presents the flowchart outlining the search and selection process of the studies included in this review. A flowchart based on the PRISMA guidelines was used to carry out this task [26,27].

### 3.2. Study Characteristics

Among the nine studies included in the review, four were RCTs [29,32,33,34], while the remaining five were crossover clinical trials [20,28,30,31,35]. The total follow-up period across all studies ranged from 21 days to 13 months. A total of 351 participants were included, the majority of whom were women due to the higher prevalence in this gender. The mean age varied between 50.7 ± 8.7 years and 69.1 ± 4 years. All studies included adults. Custom-made and intervention orthoses were used to compare their effectiveness in terms of pain, functional limitation, disability [28,29,30,31,32,33], kinematic parameters and plantar pressure distribution [20,28,35], as well as balance and walking ability [29,31,32] and improvements in quality of life with placebo FOs [33,34]. These orthoses were used either alone or in combination with personalised silicone orthoses [35] (Table 1).

### 3.3. Main Results

Several studies have demonstrated that FOs are effective in reducing pain and improving function in patients with rheumatoid arthritis RA [28,29,30,32]. However, other authors have found improvements in pain but not in function [33,34]. Some studies have shown biomechanical improvements with the use of FOs [20,28,29,32], while Maddali Bongi et al. [28] did not observe changes in biomechanical function. No significant improvements in quality of life were reported [32,33].

The studies reviewed are summarised below. 

Maddali Bongi et al. [28] conducted a crossover study comparing customised FOs, with and without silicone orthoses. They used 5 mm-thick breathable polypropylene terephthalate FOs with double layers. The combination of FOs and silicone orthoses significantly reduced pain and disability, increased the plantar contact area, and improved foot function.

Gibson et al. [35] carried out a controlled clinical trial comparing FOs produced using a 3D computer-aided design with standard polypropylene FOs. The 3D FOs manufactured via selective laser sintering and fused lay-up moulding showed no statistically significant difference in activity levels or symptoms compared to standard FOs. However, patients reported improvements in perceived efficacy, comfort, and fit of the orthotic device.

Gatt et al. [30] assessed the efficacy of FOs of different hardnesses—subortholen and ethyl vinyl acetate—in a crossover study involving participants with RA and chronic ankle involvement.. These FOs effectively reduced pain, functional limitation, and disability, making them suitable for managing the rheumatoid foot in advanced stages of the disease.

Moreira et al. [32] performed a RCT to evaluate the effectiveness of FOs with metatarsal pads and medial arch support in patients with RA. The custom-made FOs were constructed from 5 mm-thick ethyl vinyl acetate with a hardness of 35 ± 5 Shore A and a density of 0.160 g/cm^3^. They reduced pain and improved foot function, with greater improvements observed with longer wear times.

Rome et al. [34] conducted a RCT examining the clinical cost-effectiveness of customised versus plain FOs in patients with RA. The personalised FOs, made from high-density ethyl vinyl acetate, 20 mm-thick, were designed to address hindfoot valgus. They demonstrated greater efficacy in reducing pain and disability than plain FOs, despite the increased fabrication time and cost.

Linberg and Mengshoel [31] conducted a crossover study and found that thin, prefabricated, customised FOs provided immediate relief from forefoot pain during walking in patients with milder RA. These FOs were 4 mm-thick, made from malleable plastic with a textile top layer, and supported the transverse and longitudinal arches of the foot.

Reina-Bueno et al. [33] conducted a RCT comparing personalised FOs with placebo FOs on pain, disability, foot function, and quality of life. The customised FOs featured a 2 mm polypropylene layer under the metatarsal heads, 30 Shore A polyethylene foam, 5 mm ethyl vinyl acetate stabilising elements in the heel, and a 30 Shore A sub-digital ridge. Significant improvements were observed in foot pain, though there were no notable changes in disability, function, or quality of life.

Gaino et al. [29] conducted a RCT comparing the use of custom-made FOs against no intervention. The FOs were made of ethyl vinyl acetate and featured a medial arch support of approximately 10–12 mm and a metatarsal pad of 5–6 mm in thickness. Patients who used the FOs experienced improvements in function, mobility, and balance.

Simonsen et al. [20] performed a cross-sectional study comparing prefabricated and personalised FOs with a control group. Prefabricated FOs had limited effects on gait. In contrast, customised FOs, which included medial arch support and were made of ethyl vinyl acetate, resulted in significant improvements in gait, demonstrating their superiority over both the prefabricated and control groups.

Despite the recognised importance of proper footwear and its relationship with foot orthoses, research in this area remains limited. No studies meeting the selection criteria specifically related to any type of footwear therapy in individuals with RA were identified.

### 3.4. Studies’ Quality Assessment

**Figure 2:**
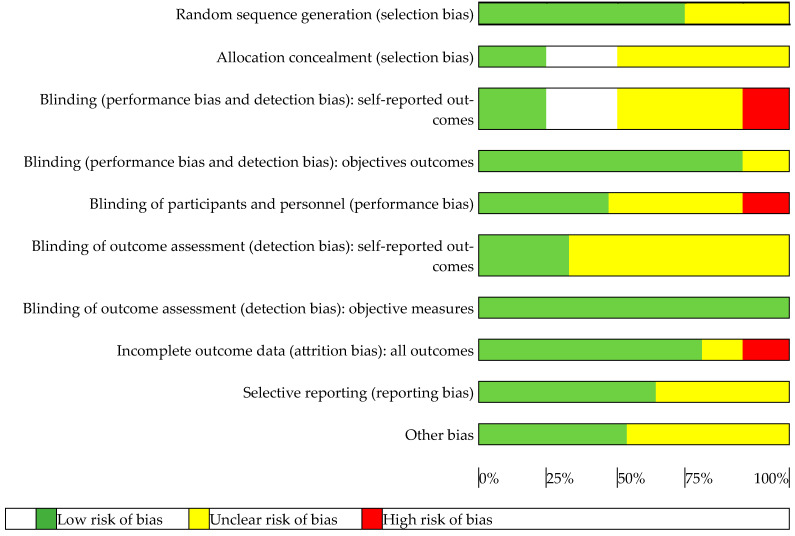
Risk of bias in the included studies.

**Figure 3:**
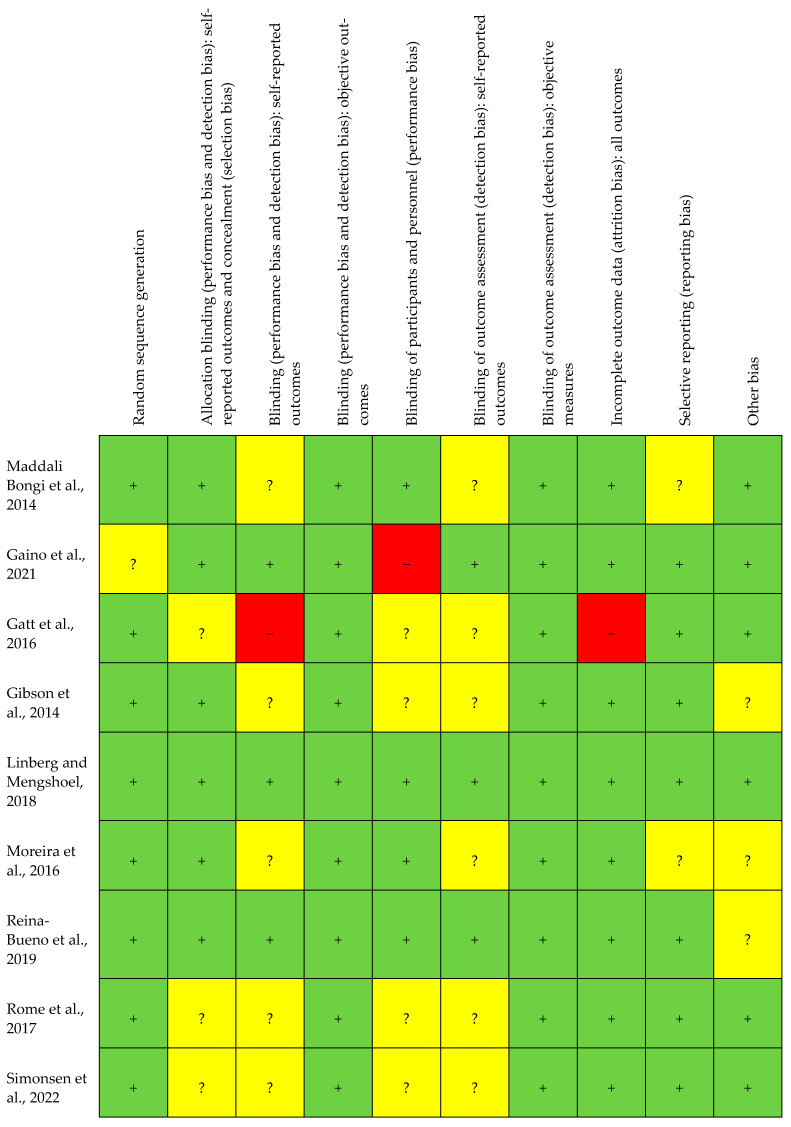
Bias assessment of the selected studies: individual analysis [20,28,29,30,31,32,33,34,35]. Symbols and colours represent the risk of bias assessment for individual studies: ‘+’ (green) indicates low risk of bias, ‘?’ (yellow) indicates unclear risk of bias, and ‘-’ (red) indicates high risk of bias.

The methodology of the quality assessment based on Review Manager (RevMan), web version, is summarised in Figure 2 and Figure 3. In the RevMan scale, all studies exhibited a low risk of bias [36]. Figure 2 describes the risk of bias in the included studies, and Figure 3 shows the assessment of the selected studies.

## 4. Discussion

RA is a chronic, progressive inflammatory disease with systemic effects, and the feet are among the most frequently affected areas, with an incidence of up to 90% as the disease progresses [38,39]. Moreover, FOs and appropriate footwear are considered suitable therapeutic options for managing foot disorders in patients with RA [38,39]. This systematic review evaluated the impact of treatment with FOs and footwear on patients with RA. Notably, no recent research has been found regarding the use of footwear as a therapeutic option. In terms of treatment with FOs, the key aspects studied include pain, self-perceived foot function, biomechanical parameters of gait, and quality of life. Although the studies included in this review exhibited certain methodological limitations, this type of treatment is still considered effective for patients with RA.

In this context, the studies selected for this review underscore the effectiveness of FOs in reducing pain and improving self-perceived foot function; on the other hand, debates persist regarding the types of FOs, the materials used, and the duration of use [28,29,30,32]. For instance, Maddali Bongi et al. [28] demonstrated that combining polypropylene FOs with customised silicone orthotics led to significant improvements in pain and functional capacity, measured by the Foot Function Index (FFI). Additionally, Gatt et al. [30] also employed semi-rigid FOs to reduce rearfoot mobility, yielding similar results. Gaino et al. [29] adopted a more contemporary approach by evaluating personalised ethylene vinyl acetate FOs. Their study emphasised the importance of adapting to current technological advancements and materials in the prescription of FOs [29]. Moreira et al. [32] provided evidence of the long-term effectiveness of customised FOs featuring medial arch support and metatarsal pads, demonstrating improvements in both walking and resting abilities at 180 days.

Conversely, some authors, including Reina-Bueno et al. [33] and Rome et al. [34], noted that semi-rigid FOs alleviated pain but did not demonstrate significant changes in patient-reported function. These findings highlight the need for further research to better understand the relationship between the use of FOs and patients’ walking ability. Similar assertions can be drawn from the meta-analysis conducted by Conceição in 2015 [22].

Additionally, custom-made FOs have been extensively shown to be effective in the prevention of diabetic foot complications [40]. The use of handmade semi-rigid FOs, combined with materials designed for shock absorption in the forefoot, has demonstrated symptomatic improvement in patients with other autoimmune disorders, such as lupus [41], as well as connective tissue disorders, such as Ehlers–Danlos syndrome [42] and other connective tissue diseases [43].

However, the 2019 review by Tenten-Diepentmaat et al. [23] summarised the literature on the comparative effectiveness of FOs for treating various foot problems in patients with RA. They found that the use of soft materials led to an immediate reduction in forefoot plantar pressure compared to orthoses made from semi-rigid materials [23]. The selection of different manufacturing techniques and materials for prescribing FOs in both intervention and control groups complicates the establishment of which type of insole may offer the best cost–benefit ratio for these patients [23].

The meta-analysis led by Gijon-Nogueron in 2018 [38] concluded that FOs alleviate pain and disability in the feet of individuals with RA in both the short and medium term. However, they found no significant differences compared to the control group. As mentioned in the article itself, this may be attributed to the small sample size or the insufficient sensitivity of the FFI questionnaire, which was used by most authors to assess foot function.

In summary, the clinical implication of this review is that personalised FOs can be recommended as a complementary treatment for patients with RA. Although further studies are needed to reach a consensus on the most effective type of FOs for patients with RA, customised FOs emerge as an effective intervention for reducing pain and improving self-perceived foot function in individuals with RA.

It is noteworthy that previous systematic reviews have primarily evaluated the effects of FOs on disability and pain [22,38]. This review also assessed the effects of this type of treatment on biomechanics and quality of life.

Specifically, regarding the biomechanical effects of gait, the findings varied. Maddali Bongi et al. [28] utilised podobarometers and the GAIT Rite System to assess plantar pressures and spatial and temporal parameters of gait, demonstrating that FOs reduced plantar pressures; however, no significant changes were identified in the kinematic parameters. Conversely, Gibson et al. [35] advanced the creation of personalised orthoses using 3D-printing technologies, showing significant biomechanical improvements, particularly in reducing the rearfoot eversion moment.

Simonsen et al. [20] explored the kinetics and kinematics of the foot and ankle using sensors integrated into footwear, finding that personalised FOs with medial arch support reduced ankle plantar flexion and the knee angle, potentially translating to a more efficient and less painful gait. These studies suggested that customised FOs offer significant benefits; however, further research is needed to optimise their use in patients with RA in more advanced stages [20,35].

Overall, despite improvements in pain and biomechanical parameters, studies have not demonstrated significant changes in patients’ quality of life with RA [32,33]. Both Moreira et al. [32] and Reina-Bueno et al. [33] assessed quality of life using tools such as the 36-Item Short-Form Survey (SF-36) and the 12-Item Short-Form Survey (SF-12), finding no significant improvements when comparing personalised FOs to placebos. This lack of improvement may be related to the subjective and complex nature of quality of life, which is influenced by multiple factors, including emotional state and the overall impact of RA on health [33]. This underscores the need for a multidisciplinary approach to comprehensively enhance the quality of life for these patients.

The clinical implication of these findings is that personalised FOs can be recommended as a complementary treatment in the management of RA, with the aim of reducing pain and improving self-perceived foot function, as well as enhancing biomechanical parameters. Nevertheless, further studies are needed to reach a consensus on the most effective types of FOs for these patients.

The use of therapeutic footwear, custom-made shoes, and modifications to existing footwear is considered a valid option for managing RA, as outlined in recent clinical practice guidelines for these patients [23,39].

However, the lack of recent research prevents clear conclusions regarding the effectiveness of therapeutic footwear in reducing pain and improving function, balance, and walking capacity. Notably, the review conducted by Frecklington et al. [24] concluded that symptoms and disability in patients with foot and ankle arthritis decreased with the use of modified footwear (technical adaptations) and therapeutic shoes. Given their potential to influence plantar pressure distribution and foot biomechanics, further studies are needed to evaluate their impact in greater depth, to draw more robust conclusions and support the use of therapeutic footwear as a complement to FOs [24].

Although the number of RCTs and crossover studies identified was not extensive, it may be adequate considering the selection criteria focused exclusively on FOs, which generally exhibit moderate methodological quality. It is also noteworthy that the assessment of effect was recorded over a short to moderate period, with only the study by Limberg and Mengshoel [31] conducting follow-up over a year of treatment. Furthermore, it is important to analyse the reasons for adherence loss, as this may significantly influence the results. Lastly, it should be noted that most of these studies did not control for factors that modulate pain or disability in the foot, such as the pharmacological therapy prescribed to patients, as evidenced by the recent work of Gamez-Guijarro et al. [44].

Concisely, considering the results of this review, it is evident that future studies are needed to compare the efficacy of different types of FOs as well as therapeutic footwear, given the potential of these treatments to improve both the signs and symptoms of RA. Additionally, it would be valuable to include quality of life assessments and qualitative research to evaluate the long-term effectiveness of the treatment and its impact on the daily lives of these patients.

## 5. Conclusions

Customised FOs stand out as a promising intervention for the treatment of RA, demonstrating significant improvements, such as reduced pain, decreased disability, and enhanced balance in adult patients. However, despite the observed benefits in specific areas, the use of FOs is not conclusively associated with a significant improvement in patients’ quality of life, suggesting that additional factors may influence the overall perception of well-being. The importance of proper fitting, particularly with effective arch and metatarsal pad support, is emphasised as a key factor in maximising the therapeutic efficacy. Despite these benefits, the findings indicated that certain functional aspects may not experience substantial improvement with the use of FOs. It is also important to note that, although FOs positively impact balance, the evidence remains inconclusive regarding significant improvements in walking ability in patients with RA. These findings underscore the importance of considering multiple factors when assessing the therapeutic impact of FOs in adults with RA. Notably, no recent studies from 2013 to September 2024 explored the impact of footwear on individuals with rheumatoid arthritis.

## Figures and Tables

**Table 1:** Main characteristics of the included studies.

	Participants	Type of Study	Average Age (Years)	Duration of Study	Intervention	Measurements	Results Measured
Maddali Bongi S. et al., 2014 [28] (Italy)	24 *	Clinical crossover trial	69.1 ± 4	60 days	Group A: Insoles without silicone orthoses in the first 30 days and polypropylene terephthalate insoles with silicone orthoses in the next 30 days. Group B: Insoles with silicone orthoses for the first 30 days and insoles without silicone orthoses for the next 30 days.	Foot Function Index; Baropodometric; GAIT Rite System.	- Pain, disability, and functional limitation. - Plantar contact areas and pressures in upright position. - Spatiotemporal gait parameters.
Gaino J.Z. et al., 2021 [29] (Brazil)	Initial: 94 Final: 81	RCT	57.6 ± 10.6	4 weeks	Control Group: Did not receive any type of intervention. Intervention Group: Received treatment with tailor-made FOs.	Foot Function Index; Berg Equilibrium Scale; Timed Up and Go Test.	- Pain, disability, and functional limitation. - Ability of the subject to maintain balance in 14 movements. - Mobility, balance, walking ability, and risk of falls.
Gatt A. et al., 2016 [30] (United Kingdom)	Initial: 10 Final: 9	Clinical crossover trial	52.2 ± 9.1	3 months	First Intervention: 5 patients were treated with FOs made of subortholen and 4 patients with FOs using ethyl vinyl acetate for the cover.	Foot Function Index; Ritchie Articular Index.	- Pain, disability, and functional limitation.
Gibson K.S. et al., 2014 [35] (Scotland)	Initial: 16 Final: 15	Clinical crossover trial	50.7 ± 8.7	21 days	First FOs Set: Using selective laser sintering, 7 days. Second FOs set: Using fused deposition modelling, 7 days. Third Intervention: Using standard set FOs, 7 days	Likert numerical rating scale.	- Self-reported comfort, fit and efficacy of the orthotic device, symptoms, and activity levels.
Linberg B.H. and Mengshoel A.M., 2018 [31] (Norway)	21 *	Clinical crossover trial	54 ± 11	12 months	First Intervention: Half received FOs and half did not. Second Intervention: The first half would not receive FOs, and the second half would.	6-Min Walk Test; Visual Analogue Scale; Borg Category Ratio Scale.	- Walking ability and distance travelled by each individual after 6-Min Walk Test. - Intensity of pain induced by walking with/without FOs.- Effort after the test.
Moreira E. et al., 2016 [32] (Brazil)	Initial: 86 Final: 80	RCT	Experimental Group: 53.3 ± 8 Control Group: 52.2 ± 9	6 months	Experimental Group: Intervention insoles.Control Group: Ethyl vinyl acetate cover placebo insole.	Ethylene vinyl acetate; Health Assessment Questionnaire; Foot Function Index and Foot Health Status Questionnaire; Short Health Survey; 6-Min Walk Test; Likert scale.	- Intensity of pain induced by walking with/without FOs. - Pain, disability, and functional limitation. Global foot function. - Assessment of general health. - Distance travelled by the individual in 6 minutes. - Patients’ perceived effect of wearing the FOs.
Reina-Bueno M. et al., 2019 [33] (Spain)	Initial: 68 Final: 53	RCT	59.2 ± 11.4	3 months	Group A: Custom-made FOs. Group B: Placebo, flat, cushioning insoles.	Foot Function Index; Ethylene vinyl acetate; Manchester Foot Pain and Disability Index; 12-Item Short-Form Health Survey.	- Pain, disability, and functional limitation. - Intensity of pain induced by walking with/without FOs. - Disability related to foot pain. - Quality of life.
Rome K. et al., 2017 [34] (New Zealand)	Initial: 41 Final: 29	RCT	62 ± 10	16 weeks	Group A: FOs made to measure. Group B: Simple cushioning insole adaptable to the shoe.	Foot Function Index; HRQoL; QALY.	- Pain, disability, and functional limitation. - Cost-utility considering health-related quality of life. - Health gain expressed as quality-adjusted life years.
Simonsen M.B. et al., 2022 [20] (Denmark)	27 *	Clinical crossover trial	59.2 ± 11.4	13 weeks	Six ground walking tests were carried out using two types of custom-made FOs and one control FOs. 1. FOs with metatarsal pads. 2. FOs with medial arch support. 3. FOs latex control.	Normal pressure; motion capture; inverse dynamics; inverted dynamics.	- Pressure at the FOs interface via the plantar pressure sensor in the shoe. - Motion capture using Qualisys Track version 2018.8.1. - Reverse dynamics using Any Body Modelling System version 7.2.3.

* For studies where both initial and final sample sizes are reported, the values are presented as ‘Initial: X’ and ‘Final: Y’. Studies marked with an asterisk (*) did not report both initial and final sample sizes. Abbreviations: foot orthoses: FOs; randomised controlled trial: RCT; health-related quality of life: HRQoL; quality-adjusted life years: QALY.

## Data Availability

The raw data supporting the conclusions of this article will be made available by the main author (J.M.C.-S.) upon request (joscabsan3@alum.us.es).

## Supplementary Materials

The following supporting information can be downloaded at: https://www.mdpi.com/article/10.3390/healthcare12202017/s1.

## Appendix A. Original and Updated Search Strategies

PubMed

- Filters (original search): Last 10 years; adult 19+ years; language: Spanish and English; article type: EC and ECA.
- Filters (updated search): January 2023–September 2024; adults 19+ years; language: Spanish and English; article type: RCT and crossover trials.
- Search strategy and results obtained:
  - (“Arthritis Rheumatoid” OR “Rheumatoid, Arthritis”) AND (foot OR (“foot pain” OR “foot deformity”)) AND (treatment OR therapy). Original search: 33 results. Updated search: 0 results.
  - (“Arthritis Rheumatoid” OR “Rheumatoid, Arthritis”) AND (foot OR (“foot pain” OR “foot deformity”)) AND ((insole OR “foot orthoses”) OR shoes OR (footwear)): Original search: 11 results. Updated search: 0 results.

Scopus

- Filters (original search): Last 10 years; language: Spanish and English; type of document: article.
- Filters (updated search): January 2023–September 2024; language: Spanish and English; type of document: article.

Limit to exact keyword: “Adult”, “Human”, “Young Adult”, “Foot pain”, “Pain”, “Patient Satisfaction”.

Exclude exact keyword: “Surgery”, “Foot Surgery”, “Postoperative Complication”, “Methotrexate”, “Antirheumatic Agents”, “Etarnecept”, “Hydroxichloroquine”, “Prednisolone”, “Corticosteroid”, “Nonsteroid Anti-inflammatory Agent”, “Infliximab”, “Osteotomy”, “Glucocorticoid”, “Drug Efficacy”, “Tocilizumab”, “Leflunomide”, “Abatacept”, “Ankle Arthroplasty”, “Rituximab”, “Arthropathy”, “Ankle Arthrodesis”, “Arthroplasty, Replacement Ankle”, “Arthrodesis” and “Hand Radiography”.

- Search strategy and results obtained:
  - (“Arthritis Rheumatoid” OR “Rheumatoid, Arthritis”) AND (foot OR (“foot pain” OR “foot deformity”)) AND (treatment OR therapy). Original search: 156 results. Updated search: 0 results.
- Filters (original search): Last 10 years; language: Spanish and English; type of document: article.
- Filters (updated search): January 2023–September 2024; language: Spanish and English; type of document: article.

Limit to exact keyword: “Human”, “Rheumatoid Arthritis”, “Foot Orthoses”, “Foot”, “Foot pain”, “Pain”, “Shoe”, “Randomized Controlled Trial”, “Podiatry”, “Biomechanics”, “Quality of life”, “Dally Life Activity”, “Orthopedic Shoe”, “Patient Satisfaction”, “Footwear”, “Pain Assessment”, “Patient-reported Outcome”, “Plantar Pressure”, “Young Adult”, Insole”, “Orthosis”, “Orthotic Devices” and “Foot Orthotics”.

Exclude exact keyword: “Methotrexate”, “Disease Modifying Antirheumatic Drug”, “Juvenile Rheumatoid Arthritis”, “Osteotomy”, “Surgery”, “Child”, “Surgical Technique”, “Adalimumab”, “Etarnecept”, “Foot Surgery”, “United Kingdom”, “Arthrodesis”, “Heel”, “Ankylosing Spondylitis” “Arthritis, Psoriatic”, “Arthroplasty”, “Physiotherapy”, and “Gout”.

- Search strategy and results obtained:
  - (“Arthritis Rheumatoid” OR “Rheumatoid, Arthritis”) AND (foot OR “foot pain” OR “foot deformity”) AND (insole OR “foot orthoses” OR shoes OR footwear). Original search: 50 results. Updated search: 0 results.

CINALH

- Filters (original search): Last 10 years; language: English and Spanish; type of document: clinical trial and randomised controlled trial; age: adult (19–44 years), middle aged (45–64 years), and elderly (65+ years, 80 and over).
- Filters (updated search): January 2023–September 2024; language: Spanish and English; type of document: clinical trial and randomised controlled trial; age: adult (19–44 years), middle aged (45–64 years), and elderly (65+ years, 80 and over).
- Search strategy and results obtained:
  - (“Arthritis Rheumatoid” OR “Rheumatoid, Arthritis”) AND (foot OR (“foot pain” OR “foot deformity”)) AND (treatment OR therapy). Original search: 19 results. Updated search: 2 results.
  - (“Arthritis Rheumatoid” OR “Rheumatoid, Arthritis”) AND (foot OR (“foot pain” OR “foot deformity”)) AND ((insole OR “foot orthoses”) OR shoes OR (footwear)). Original search: 6 results. Updated search: 2 results.

Web of Science

- Filters (original search): Last 10 years; language: English and Spanish; type of document: article; exclude: Pharmacology Pharmacy or Oncology and Surgery; citation topics micro: 1.106.188 Rheumatoid Arthritis.
- Filters (updated search): January 2023–September 2024; language: English and Spanish; type of document: article; exclude: Pharmacology Pharmacy or Oncology and Surgery; citation topics micro: 1.106.188 Rheumatoid Arthritis.
- Search strategy and results obtained:
  - (“Arthritis Rheumatoid” OR “Rheumatoid, Arthritis”) AND (foot OR (“foot pain” OR “foot deformity”)) AND (treatment OR therapy): Original search: 139 results. Updated search: 0 results.
  - (“Arthritis Rheumatoid” OR “Rheumatoid, Arthritis”) AND (foot OR (“foot pain” OR “foot deformity”)) AND ((insole OR “foot orthoses”) OR shoes OR (footwear)). Original search: 6 results. Updated search: 1 result.

Dialnet

- Filters (original search): 10 years; language: English and Spanish; type of document: article.
- Filters (updated research): January 2023–September 2024; language: English and Spanish; type of document: article.
- Search strategy and results obtained:
  - (“Arthritis Rheumatoid” OR “Rheumatoid, Arthritis”) AND (foot OR (“foot pain” OR “foot deformity”)) AND (treatment OR therapy). Original search: 7 results. Updated search: 1 result.
  - (“Arthritis Rheumatoid” OR “Rheumatoid, Arthritis”) AND (foot OR (“foot pain” OR “foot deformity”)) AND ((insole OR “foot orthoses”) OR shoes OR (footwear)). Original search: 5 results. Updated search: 0 results.
